# The value of innovation: association between improvements in survival of advanced and metastatic non-small cell lung cancer and targeted and immunotherapy

**DOI:** 10.1186/s12916-021-02070-w

**Published:** 2021-09-15

**Authors:** Sreeram Ramagopalan, Thomas P. Leahy, Joshua Ray, Samantha Wilkinson, Cormac Sammon, Vivek Subbiah

**Affiliations:** 1 F. Hoffmann-La Roche Ltd, Grenzacherstrasse 124, 4070 Basel, Switzerland; 2 PHMR Ltd., Westport, Ireland; 3 Roche Products Ltd, Welwyn Garden City, UK; 4 Department of Investigational Cancer Therapeutics, Division of Cancer Medicine, UT MD Anderson Cancer Center, Houston, USA; 5 MD Anderson Cancer Network, UT MD Anderson Cancer Center, Houston, USA; 6 Clinical Center For Targeted Therapy, UT MD Anderson Cancer Center, Houston, USA; 7 Division of Pediatrics, UT MD Anderson Cancer Center, Houston, USA

**Keywords:** Lung cancer, NSCLC, Immunotherapy, Innovation, Targeted therapy

## Abstract

**Background:**

Significant improvements in mortality among patients with non-small cell lung cancer (NSCLC) in the USA over the past two decades have been reported based on Surveillance, Epidemiology, and End Results (SEER) data. The timing of these improvements led to suggestions that they result from the introduction of new treatments; however, few studies have directly investigated this. The aim of this study was to investigate the extent to which population level improvements in survival of advanced and/or metastatic NSCLC (admNSCLC) patients were associated with changes in treatment patterns.

**Methods:**

We utilized a de-identified database to select three cohorts of patients with admNSCLC: (1) patients with non-oncogene (EGFR/ALK/ROS1/BRAF) positive tumors, (2) patients with ALK-positive (ALK+) tumors, and (3) patients with EGFR-positive (EGFR+) tumors. All patients were diagnosed with admNSCLC between 2012 and 2019. Multivariable Cox models adjusting for baseline characteristics and receipt of targeted and immunotherapy were utilized to explore the relationship between these variables and changes in the hazard of death by calendar year in each cohort.

**Results:**

We included 28,154 admNSCLC patients with non-oncogene positive tumors, 598 with ALK+ tumors, and 2464 with EGFR+ tumors eligible for analysis. After adjustment for differences in baseline characteristics, the hazard of death in patients who had non-oncogene positive tumors diagnosed in 2015, 2016, 2017, 2018 ,and 2019 was observed to be 12%, 11%, 17%, 20%, and 21% lower respectively than that for those diagnosed in 2012. Upon additionally adjusting for receipt of first line or second line immunotherapy, the decrease in the hazard of death by calendar year was no longer observed, suggesting improvements in survival observed over time may be explained by the introduction of these treatments. Similarly, decreases in the hazard of death were only observed in patients with ALK+ tumors diagnosed between 2017 and 2019 relative to 2012 but were no longer observed following adjustment for the use of 1st and later generation ALK inhibitors. Among patients with EGFR+ tumors, the hazard of death did not improve significantly over time.

**Conclusion:**

Our findings expand on the SEER data and provide additional evidence suggesting improvements in survival of patients with advanced and metastatic NSCLC over the past decade could be explained by the change in treatment patterns over this period.

**Supplementary Information:**

The online version contains supplementary material available at 10.1186/s12916-021-02070-w.

## Background

Over the past 20 years, targeted inhibitors of both the ALK and EGFR oncogene have been successfully developed and approved for use in patients with non-small cell lung cancer (NSCLC) testing positive for these oncogenes [1]. More recently, immunotherapies have also emerged as an efficacious treatment option in individuals with tumors not testing positive for these driver oncogenes or among those with tumors testing positive for other biomarkers such as PD-L1 [2, 3]. The benefits of these innovative treatments in patients with NSCLC have been demonstrated in clinical trials and observational studies, and they have therefore been proposed as a potentially plausible explanation for the improvements in population level mortality among patients with NSCLC in the USA [4]. Despite such suggestions, little work has been done to explore whether the increases in use and incremental benefits of innovative treatments are of sufficient magnitude to explain the population level improvements reported.

As such, in this study, we sought to utilize patient level data from a large US electronic health record (EHR) database to investigate improvements in survival of patients with advanced and/or metastatic NSCLC (admNSCLC) over time and explored their relationship with changes in treatment patterns.

## Methods

### Data source

This study utilized the deidentified Flatiron Health EHR-derived database. It is a US-wide longitudinal database that is comprised of patient-level structured and unstructured data, curated via technology-enabled abstraction [5]. During the study period, the de-identified data originated from approximately 280 US cancer clinics (~ 800 sites of care). The majority of patients in the database originate from community oncology settings; relative community/academic proportions may vary depending on study cohort. The data source has been described in detail elsewhere [5, 6].

### Study population

All patients with admNSCLC diagnosed between 2012 and 2019 and who had complete historical treatment data, i.e., no gaps of 90 days or more between the date of advanced/metastatic diagnosis and start of observed structured data, were identified. To ensure analyses were carried out in populations with similar treatment recommendations, this population was stratified into three sub-cohorts based on their oncogene positivity: (1) patients whose tumors were non-oncogene (EGFR/ALK/ROS1/BRAF) positive, (2) patients who had ALK-positive (ALK+) tumors, and (3) patients with EGFR-positive (EGFR+) tumors. Patients with missing oncogene data were classified in the non-oncogene positive group given the expectation that the majority of such patients would have oncogene negative tumors. Baseline characteristics (age, sex, smoking status, stage at initial diagnosis, time from diagnosis to 1st line treatment, histology, race) were extracted for each patient in addition to information on treatment with immunotherapies, ALK inhibitors, and EGFR inhibitors (list of specific therapies provided in Additional file 2). The raw data for the race variable included an others category and due to small numbers, patients with reported race of Asian or Hispanic/Latino were included in the others category.

### Analysis

Descriptive statistics for population characteristics were calculated. For each sub-cohort, complete case multivariable Cox models adjusting for the baseline characteristics and/or treatments described above were utilized to estimate hazard ratios describing the hazard of death by calendar year. The treatment variable was defined using the treatments that patients received over their entire follow-up and adjusted for at baseline. For models in which the non-proportional hazards assumption was determined not to hold [7], weighted Cox regression was applied using Prentice weights with a censoring correction and robust variance estimation to estimate averaged hazard ratios [8]. Follow-up was measured from the date of admNSCLC diagnosis until death or the latest of treatment and last record. A sensitivity analysis was conducted to assess the sensitivity to potential misclassification, where patients who received an ALK or EGFR targeted treatment but for whom the ALK and EGFR oncogene status of their tumor was missing were reclassified as either ALK+ or EGFR+ respectively. In addition, another sensitivity analysis was conducted where a missing/not-recorded category was included for covariates with missing or not-recorded data as well as adjusting for other peripheral covariates and covariates that had significant missingness/under-recording, namely practice type, insurance type, Eastern Cooperative Oncology Group performance status (ECOG), and site of metastases.

R version 4.0.0 was used for all analyses.

## Results

### Non-oncogene positive cohort

**Fig. 1:**
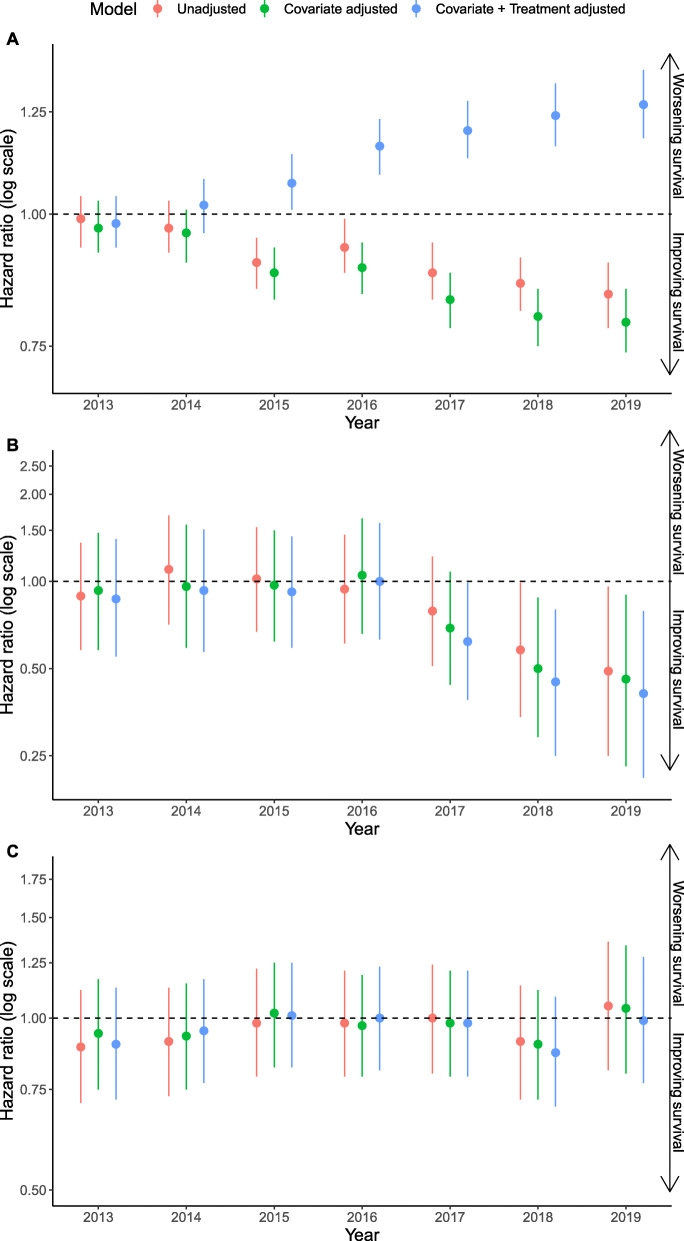
Hazard ratios for death in non-oncogene positive (**A**) advanced and/or metastatic NSCLC, (**B**) ALK-positive and (**C**) EGFR-positive patients in years 2013 to 2019 relative to 2012, unadjusted, adjusting only for differences in baseline characteristics and adjusting for both baseline characteristics and (**A**) immunotherapy use, (**B**) ALK inhibitor use and (**C**) EGFR inhibitor use

**Fig. 2:**
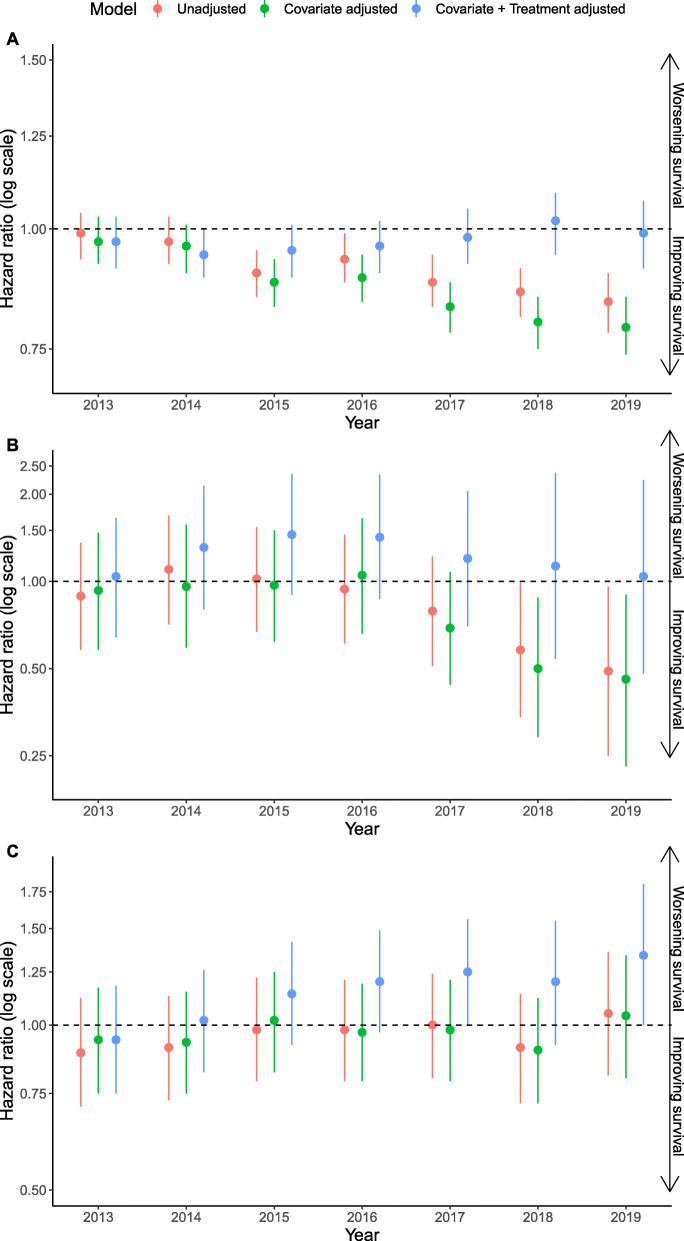
Hazard ratios for death in non-oncogene positive (**A**) advanced and/or metastatic NSCLC, (**B**) ALK-positive and (**C**) EGFR-positive patients in years 2013 to 2019 relative to 2012, unadjusted, adjusting only for differences in baseline characteristics and adjusting for both baseline characteristics and (**A**) first and/or second line immunotherapy, (**B**) first and/or second/third generation ALK inhibitor use, and (**C**) first/second and/or third generation EGFR inhibitor use

We identified 32,458 patients with non-oncogene positive tumors who were eligible for the study, of which 28,154 patients had complete data and relevant treatment for analysis. Missingness among all covariates was less than 5% with time to first-line treatment and group stage the covariates contributing the majority of the missingness. One year survival probability in this cohort increased over time from 50% in 2012 to 57% in 2019 (see Additional file 1). The hazard of death adjusting for baseline characteristics in non-oncogene positive patients diagnosed in 2015, 2016, 2017, 2018, and 2019 was observed to be 12%, 11%, 17%, 20%, and 21% lower respectively than that in those diagnosed in 2012 (Fig. 1A). After additionally adjusting for receipt of immunotherapy in any line of therapy where the treatment variable was split into four categories, approved and unapproved immunotherapies, approved immunotherapies only, unapproved immunotherapies only, and other treatments, the hazard of death was observed to increase by calendar year (Fig. 1A and Additional file 2). Given that first line immunotherapy may be expected to have a greater impact on outcomes than second line immunotherapy, the relationship was explored further in post hoc analyses adjusting for the specific regimen of treatment received across first line or second line, in line with US Food and Drug Administration (FDA) approvals. The hazard of death was stable across calendar year in this analysis (Fig. 2A and Additional file 2).

### ALK+ cohort

We identified 690 patients who had ALK+ tumors and were eligible for the study, of which 598 patients had complete data and had received approved treatments for analysis. Missingness among all covariates was less than 5% with time to first-line treatment contributing the majority of the missingness. One year survival probability increased towards the end of the study period, from 72% in 2016 to 85% in 2019 (see Additional file 1). After adjustment for differences in baseline characteristics, significant decreases in the hazard of death relative to 2012 were observed in patients who had ALK+ tumors diagnosed between 2018 and 2019 (Fig. 1B). These decreases in the hazard of death remained following additional adjustment for the use of ALK inhibitors (Fig. 1B and Additional file 2). Given the timing of the change in hazard ratio observed we carried out a post-hoc analysis in which the treatment variable accounted separately for use of first generation and second/third generation ALK use. Decreases in the hazard of death were smaller in magnitude in this model (Fig. 2B and Additional file 2).

### EGFR+ cohort

We identified 2926 patients who had EGFR+ tumors and were eligible for the study, of which 2464 patients had complete data and had received approved treatments for analysis. Missingness among all covariates was less than 5% with time to first-line treatment and group stage the covariates contributing the majority of the missingness. One year survival probability was observed to decrease marginally over the study period (see Additional file 1) and in the model adjusting for baseline characteristics the hazard of death did not improve significantly over time (Fig. 1C). The post hoc analysis where the treatment variable was divided by generation of EGFR inhibitor demonstrated a similar trend to the main analysis (Fig. 2C and Additional file 2).

### Sensitivity analyses

The sensitivity analysis assessing potential for misclassification found in both the main analysis and post hoc analysis that the results for the non-oncogene positive cohort and ALK+ cohort to be robust to assumptions around the classification of patients with missing oncogene status but found some sensitivity in the results in the EGFR+ cohort, whereby the previously stable trends were replaced with a marginal decreasing trend of the hazard of death (Additional file 3). Results across all cohorts were robust to sensitivity analysis in which variables with a high proportion of missingness were included in the model and missing categories were used instead of a complete case analysis (Additional file 3).

## Discussion

Our findings highlight the improvement in survival of the admNSCLC patient population and suggest such improvements may be a result of changes in treatment over the past decade.

Our descriptive analyses build on recent work in broader populations of lung cancer and NSCLC patients [4, 9], demonstrating population level improvements in survival of subgroups of patients with admNSCLC over time which align well with the timing of the introduction of targeted and immunotherapies. However, by utilizing patient level EHR data on treatment and outcomes, we have been able to go further than previous studies, demonstrating that the changes in treatment patterns may plausibly explain the observed improvements in survival.

Significant improvements in survival among patients with non-oncogene positive tumors were observed from 2015 onwards, a timing that aligns with the earliest approvals of immunotherapies for this population. Adjustment for treatment in our main analysis provided an increase in the hazard of death by calendar year, suggesting that, independent of treatment, survival may have got worse over time. Adjusting for treatment in a more detailed manner in post-hoc analyses indicated that, independent of treatment, survival remained stable over time, suggesting that the categorization of treatment patterns in our main analysis may not have been granular enough to appropriately capture the relationship between treatment patterns, calendar time, and the outcomes. Notably, this post-hoc analysis suggests that changes in treatment pattern over time could explain the survival improvements observed.

Our main analysis that accounted for ALK inhibitors as a single class of drugs suggested treatment was unable to fully account for the improvements in survival observed among patients who had ALK+ tumors. The significant improvements in survival among patients with ALK+ tumors were observed between 2017 and 2019, a timing that aligns with the approval and introduction of second and third generation ALK inhibitors between 2017 and 2019. Our post-hoc analysis driven by this observation, in which we separated first and second/third generation ALK inhibitors into separate categories suggested that changes in treatment patterns over time could fully account for the survival improvements observed.

The absence of an improvement in survival among patients who had EGFR+ tumors is notable given we observed a large increase in the use of second and third generation EGFR inhibitors over our study period. Notably, the overall proportion of patients receiving any EGFR inhibitor (i.e., 1st/2nd/3rd gen) remained relatively stable over the study period suggesting that the incremental benefit of 3rd generation over 1st and 2nd generation products may not have translated into a large enough population level benefit over time for us to have the power to identify it in this study.

Patients with missing results for oncogene status were grouped into the non-oncogene positive cohort in our analyses; therefore, the impact of misclassification on our results must be considered. If these patients had ALK+ or EGFR+ tumors, their treatment patterns may have been inappropriately captured in our analyses thereby impacting the extent to which their survival over calendar time could be linked to treatment. Notably, findings remained relatively stable in sensitivity analyses investigating potential misclassification of patients with ALK+ and EGFR+ tumors into this population.

While we have been able to utilize patient level data to explore the relationship between treatment, calendar time, and outcomes, our data are observational in nature, and it is possible that other factors not fully captured in the main analysis may confound the relationships observed. Our sensitivity analyses including additional covariates provides some reassurance in this regard; however ,other changes that have occurred over the period in question may confound the associations observed; this might include broad changes in the healthcare system or in the standard of care of patients. Notably, mortality rate in the general population in the USA remained relatively constant over the period under study [10] while the classification of calendar time into annual periods overcomes additional challenges that can be encountered when comparing outcomes over calendar time, such as the impact of seasonality.

The small numbers of patients treated with some products led us to categorize treatments into classes and, in some cases, combine two classes of drug. While we have sought to group treatments in a logical manner, products within categories may have differing efficacy potentially introducing residual confounding. The short follow up available for patients diagnosed in later years may limit the power to detect differences in the patient populations with ALK+ and EGFR+ tumors given the relatively long survival in these groups. The post hoc nature of the analyses underlying a number of our key findings must also be considered. Finally, we classified patients into time constant treatment groups from baseline based on a pattern of treatments observed post-baseline. This may introduce immortal time biases, for example, owing to the fact patients receiving second line treatment had to survive long enough to receive such a treatment. In that regard, while we have provided some discussion linking the timing of improvements to the introduction of specific drugs, it is important to note that our study did not directly investigate the comparative effectiveness of different treatments or treatment classes and as, outlined above, the limitations of the study preclude one from drawing any such causal inferences from our results. Rather our study sought to explore whether changes in treatment over time may have been sufficient to explain the observed improvements in survival.

While these limitations need to be considered in interpreting our findings, we believe the fact we have demonstrated our findings across subgroups of the admNSCLC patient population, in which changes in the treatment paradigm have occurred at different points in time, suggests that changes in pharmacological treatment are as plausible an explanation for the findings as any of these other potential factors. As such, we believe our work provides a meaningful step beyond previous work in this area. Future work might seek to utilize data sources which can address a number of the above limitations or carry out similar analyses within a broader population of NSCLC patients which includes earlier stage patients.

## Conclusions

Our findings expand on the SEER data and provide additional evidence suggesting improvements in survival of patients with advanced and/or metastatic NSCLC over the past decade could be explained by the change in treatment patterns over this period.

## Supplementary Information

**Additional file 1.** One year survival probability plots. Description: One year survival probability plots for each cohort.

**Additional file 2.** Treatment classification, patient characteristics and additional results. Description: Tables showing (1) treatment classification, (2) baseline characteristics and treatment regimen among admNSCLC patients by calendar year of diagnosis (2012 to 2019) stratified by biomarker status and (3) hazard ratios for overall survival among admNSCLC patients, stratified by biomarker status.

**Additional file 3.** Sensitivity analysis plots. Description: Plots of the hazard ratios from both sensitivity analyses for each population and for both the main analysis and post hoc analysis. It also includes a table of the additional covariates used in the sensitivity analysis stratified by year.

## Data Availability

The data that support the findings of this study have been originated by Flatiron Health, Inc. These de-identified data may be made available upon request and are subject to a license agreement with Flatiron Health; interested researchers should contact <DataAccess@flatiron.com> to determine licensing terms.

## Abbreviations

ALK: Anaplastic lymphoma kinase
BRAF: v-raf murine sarcoma viral oncogene homolog B1
EGFR: Epidermal growth factor receptor
EHR: Electronic health record
FDA: Food and Drug Administration
admNSCLC: Advanced/metastatic non-small cell lung cancer
NSCLC: Non-small cell lung cancer
PD-L1: Programmed death-ligand 1
ROS1: c-ros oncogene 1
SEER: Surveillance, Epidemiology, and End Results
