# Supplementary material for: The value of innovation: association between improvements in survival of advanced and metastatic non-small cell lung cancer and targeted and immunotherapy
Source: BMC Med. 2021 Sep 15;19:209. doi: 10.1186/s12916-021-02070-w (PMC8442434; doi:10.1186/s12916-021-02070-w)
Supplement: Supplementary file 1 — Additional file 1. One year survival probability plots. Description: One year survival probability plots for each cohort. [file 12916_2021_2070_MOESM1_ESM.docx]

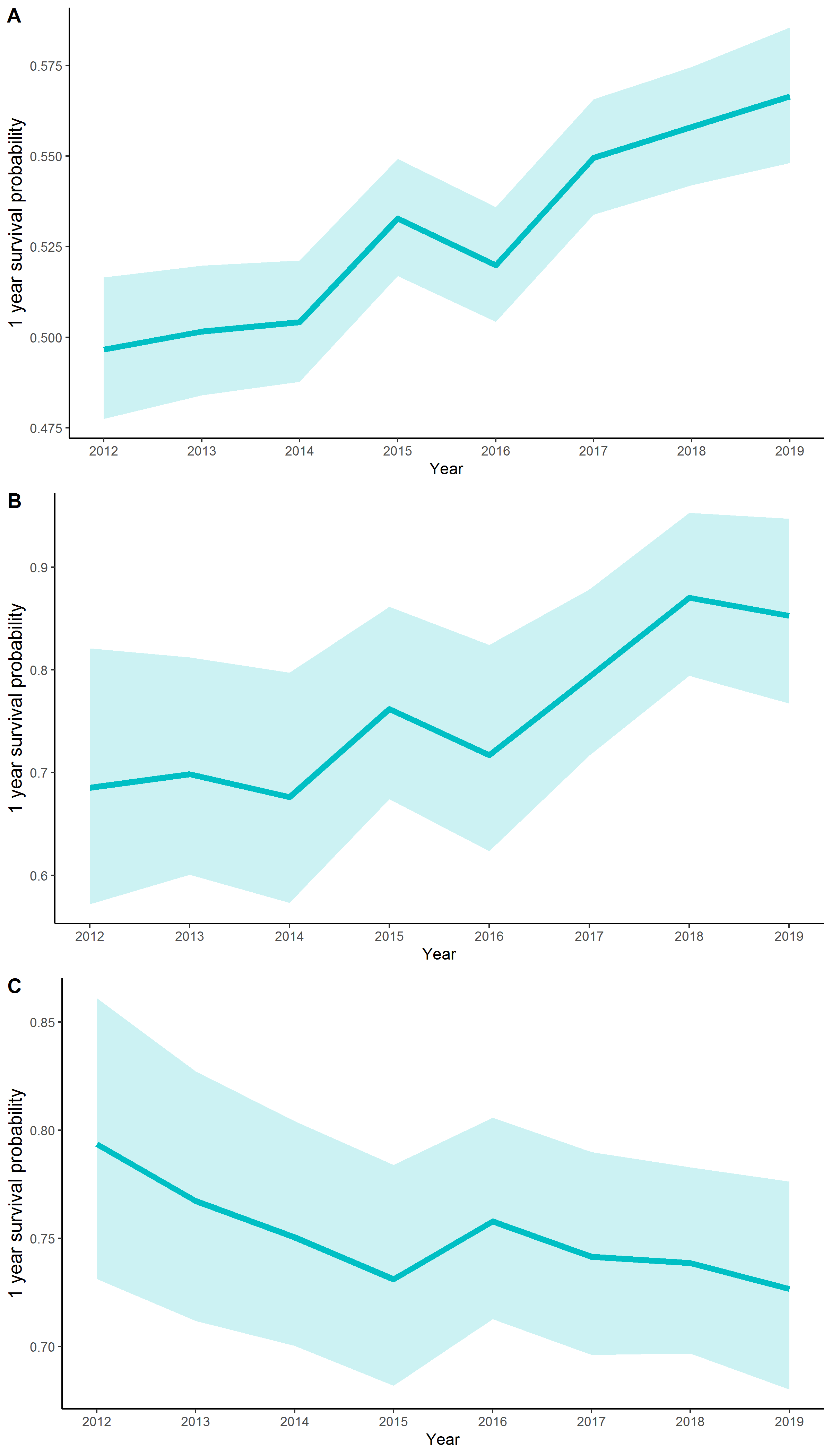


Supplementary Figure 1. One year survival probability among non-oncogene positive (A) advanced and/or metastatic NSCLC (B) ALK-positive and (C) and EGFR-positive patients diagnosed in each year from 2012 to 2019. Shaded area represents 95% confidence intervals.
