# Supplementary material for: The value of innovation: association between improvements in survival of advanced and metastatic non-small cell lung cancer and targeted and immunotherapy
Source: BMC Med. 2021 Sep 15;19:209. doi: 10.1186/s12916-021-02070-w (PMC8442434; doi:10.1186/s12916-021-02070-w)
Supplement: Supplementary file 2 — Additional file 2. Treatment classification, patient characteristics and additional results. Description: Tables showing (1) treatment classification, (2) baseline characteristics and treatment regimen among admNSCLC patients by calendar year of diagnosis (2012 to 2019) stratified by biomarker status and (3) hazard ratios for overall survival among admNSCLC patients, stratified by biomarker status. [file 12916_2021_2070_MOESM2_ESM.docx]

Supplementary Table 1. Specific treatments falling under each treatment category.

| **Immunotherapy** |
| --- |
| **First line (FDA approved)** |
| Atezolizumab |
| Cemiplimab |
| Pembrolizumab |
| Nivolumab + Ipilimumab |
| **First line (non- FDA approved)** |
| Avelumab |
| Durvalumab |
| Ipilimumab |
| Nivolumab |
| **Second line (FDA approved)** |
| Atezolizumab |
| Pembrolizumab |
| Nivolumab |
| **Second line (non- FDA approved)** |
| Avelumab |
| Cemiplimab |
| Durvalumab |
| Nivolumab + Ipilimumab |
| **ALKi** |
| **First generation** |
| Crizotinib |
| **Second generation** |
| Alectinib |
| Brigatinib |
| Ceritinib |
| **Third generation** |
| Lorlatinib |
| **EGFRi** |
| **First generation** |
| Erlotinib |
| Gefitinib |
| Icotinib |
| **Second generation** |
| Afatinib |
| Dacomitinib |
| **Third generation** |
| Almonertinib |
| Olmutinib |
| Osimertinib |
| Rociletinib |

Supplementary Table 2. Baseline characteristics and treatment regimen among mNSCLC patients by calendar year of diagnosis (2012 to 2019), stratified by biomarker status.

|  | **2012** | **2013** | **2014** | **2015** | **2016** | **2017** | **2018** | **2019** |
| --- | --- | --- | --- | --- | --- | --- | --- | --- |
|  | **Non biomarker positive mNSCLC patients** | | | | | | | |
| **Age, Mean (SD)** | 67.06 (8.79) | 67.16 (9.07) | 67.27 (9.18) | 67.27 (9.45) | 68.1 (9.33) | 68.58 (9.53) | 68.56 (9.49) | 69.12 (9.64) |
| **Sex, n(%)** |  |  |  |  |  |  |  |  |
| **Male** | 1416 (54.4%) | 1677 (53.3%) | 1954 (54.4%) | 2086 (54.5%) | 2146 (53.8%) | 2183 (55.5%) | 2102 (55.9%) | 1774 (53.6%) |
| **Female** | 1189 (45.6%) | 1472 (46.7%) | 1637 (45.6%) | 1743 (45.5%) | 1840 (46.2%) | 1747 (44.5%) | 1655 (44.1%) | 1533 (46.4%) |
| **Smoking status, n(%)** |  |  |  |  |  |  |  |  |
| **Smoker** | 2326 (91%) | 2812 (90.7%) | 3210 (90.3%) | 3476 (91.2%) | 3592 (90.6%) | 3578 (91.2%) | 3494 (93%) | 3023 (91.5%) |
| **Non-smoker** | 230 (9%) | 290 (9.3%) | 345 (9.7%) | 337 (8.8%) | 374 (9.4%) | 346 (8.8%) | 262 (7%) | 282 (8.5%) |
| **Stage, n(%)** |  |  |  |  |  |  |  |  |
| **0/I** | 182 (7%) | 223 (7.1%) | 304 (8.5%) | 304 (7.9%) | 349 (8.8%) | 365 (9.3%) | 335 (8.9%) | 295 (8.9%) |
| **II/III** | 739 (28.4%) | 868 (27.6%) | 978 (27.2%) | 1062 (27.7%) | 1130 (28.3%) | 1079 (27.5%) | 1152 (30.7%) | 1021 (30.9%) |
| **IV** | 1684 (64.6%) | 2058 (65.4%) | 2309 (64.3%) | 2463 (64.3%) | 2507 (62.9%) | 2486 (63.3%) | 2270 (60.4%) | 1991 (60.2%) |
| **Time to 1L treatment, Median (IQR)** | 1.12 (0.69 - 2) | 1.08 (0.66 - 1.84) | 1.05 (0.66 - 1.74) | 1.12 (0.69 - 1.81) | 1.08 (0.69 - 1.64) | 1.12 (0.72 - 1.71) | 1.12 (0.72 - 1.68) | 1.12 (0.72 - 1.68) |
| **Histology** |  |  |  |  |  |  |  |  |
| **Squamous cell carcinoma** | 665 (25.5%) | 850 (27%) | 932 (26%) | 1052 (27.5%) | 1095 (27.5%) | 1157 (29.4%) | 1115 (29.7%) | 1023 (30.9%) |
| **Non-squamous cell carcinoma** | 1787 (68.6%) | 2124 (67.4%) | 2467 (68.7%) | 2594 (67.7%) | 2685 (67.4%) | 2601 (66.2%) | 2466 (65.6%) | 2162 (65.4%) |
| **NSCLC histology NOS** | 153 (5.9%) | 175 (5.6%) | 192 (5.3%) | 183 (4.8%) | 206 (5.2%) | 172 (4.4%) | 176 (4.7%) | 122 (3.7%) |
| **Ethnicity** |  |  |  |  |  |  |  |  |
| **White** | 2103 (80.7%) | 2440 (77.5%) | 2775 (77.3%) | 2969 (77.5%) | 3078 (77.2%) | 3055 (77.7%) | 2920 (77.7%) | 2495 (75.4%) |
| **Black or African American** | 227 (8.7%) | 308 (9.8%) | 320 (8.9%) | 399 (10.4%) | 407 (10.2%) | 397 (10.1%) | 361 (9.6%) | 347 (10.5%) |
| **Missing/Others** | 275 (10.6%) | 401 (12.7%) | 496 (13.8%) | 461 (12%) | 501 (12.6%) | 478 (12.2%) | 476 (12.7%) | 465 (14.1%) |
| **Treatment, n(%)*** |  |  |  |  |  |  |  |  |
| **Approved and non-approved immunotherapy** |  |  |  |  |  |  |  |  |
| **1L approved IT; 2L non-approved IT** | <5 | <5 | <5 | <5 | <5 | <5 | <5 | 11 (0.3%) |
| **1L non-approved IT; 2L approved IT** | <5 | <5 | <5 | 20 (0.5%) | 34 (0.9%) | 23 (0.6%) | 17 (0.5%) | 9 (0.3%) |
| **Approved immunotherapies only** |  |  |  |  |  |  |  |  |
| **1L approved IT; 2L approved IT** | 0 (0%) | 0 (0%) | 3 (0.1%) | 2 (0.1%) | 18 (0.5%) | 134 (3.4%) | 175 (4.7%) | 126 (3.8%) |
| **1L approved IT; 2L Non-IT therapy** | 2 (0.1%) | 0 (0%) | 3 (0.1%) | 5 (0.1%) | 41 (1%) | 281 (7.2%) | 364 (9.7%) | 300 (9.1%) |
| **1L approved IT; 2L no therapy** | 3 (0.1%) | 5 (0.2%) | 12 (0.3%) | 16 (0.4%) | 145 (3.6%) | 856 (21.8%) | 1349 (35.9%) | 1625 (49.1%) |
| **1L Non-IT therapy; 2L approved IT** | 30 (1.2%) | 78 (2.5%) | 322 (9%) | 1111 (29%) | 1256 (31.5%) | 857 (21.8%) | 501 (13.3%) | 207 (6.3%) |
| **Non-approved immunotherapies only** |  |  |  |  |  |  |  |  |
| **1L non-approved IT; 2L non-approved IT** | <5 | <5 | <5 | <5 | <5 | <5 | <5 | <5 |
| **1L non-approved IT; 2L Non-IT therapy** | <5 | 5 (0.2%) | 11 (0.3%) | 74 (1.9%) | 84 (2.1%) | 64 (1.6%) | 49 (1.3%) | 17 (0.5%) |
| **1L non-approved IT; 2L no therapy** | <5 | 20 (0.6%) | 37 (1%) | 153 (4%) | 251 (6.3%) | 203 (5.2%) | 139 (3.7%) | 78 (2.4%) |
| **1L Non-IT therapy; 2L non-approved IT** | <5 | <5 | <5 | <5 | <5 | 155 (3.9%) | 361 (9.6%) | 372 (11.2%) |
| **No immunotherapy** |  |  |  |  |  |  |  |  |
| **1L Non-IT therapy; 2L Non-IT therapy** | 1239 (47.6%) | 1415 (44.9%) | 1333 (37.1%) | 875 (22.9%) | 604 (15.2%) | 340 (8.7%) | 170 (4.5%) | 104 (3.1%) |
| **1L Non-IT therapy; 2L no therapy** | 1325 (50.9%) | 1624 (51.6%) | 1866 (52%) | 1571 (41%) | 1546 (38.8%) | 1013 (25.8%) | 626 (16.7%) | 456 (13.8%) |
|  | **ALK+ mNSCLC patients** | | | | | | | |
| **Age, Mean (SD)** | 58.37 (11.68) | 59.25 (13.35) | 62.67 (12) | 61.26 (12.69) | 63.9 (10.71) | 62.15 (13.74) | 63.07 (14.29) | 63.58 (11.6) |
| **Sex, n(%)** |  |  |  |  |  |  |  |  |
| **Male** | 29 (53.7%) | 32 (43.8%) | 29 (41.4%) | 40 (49.4%) | 40 (51.3%) | 48 (48.5%) | 36 (50.7%) | 24 (33.3%) |
| **Female** | 25 (46.3%) | 41 (56.2%) | 41 (58.6%) | 41 (50.6%) | 38 (48.7%) | 51 (51.5%) | 35 (49.3%) | 48 (66.7%) |
| **Smoking status, n(%)** |  |  |  |  |  |  |  |  |
| **Smoker** | 22 (40.7%) | 32 (43.8%) | 37 (52.9%) | 42 (51.9%) | 46 (59%) | 46 (46.5%) | 36 (50.7%) | 24 (33.3%) |
| **Non-smoker** | 32 (59.3%) | 41 (56.2%) | 33 (47.1%) | 39 (48.1%) | 32 (41%) | 53 (53.5%) | 35 (49.3%) | 48 (66.7%) |
| **Stage, n(%)** |  |  |  |  |  |  |  |  |
| **0/I** | <5 | <5 | 5 (7.1%) | 6 (7.4%) | 10 (12.8%) | <5 | <5 | 5 (6.9%) |
| **II/III** | 7 (13%) | 14 (19.2%) | 13 (18.6%) | 16 (19.8%) | 20 (25.6%) | 21 (21.2%) | 10 (14.1%) | 13 (18.1%) |
| **IV** | 45 (83.3%) | 56 (76.7%) | 52 (74.3%) | 59 (72.8%) | 48 (61.5%) | 74 (74.7%) | 57 (80.3%) | 54 (75%) |
| **Time to 1L treatment, Median (IQR)** |  |  |  |  |  |  |  |  |
| **Histology** |  |  |  |  |  |  |  |  |
| **Squamous cell carcinoma** | <5 | <5 | <5 | <5 | <5 | 5 (5.1%) | <5 | <5 |
| **Non-squamous cell carcinoma** | 51 (94.4%) | 68 (93.2%) | 68 (97.1%) | 77 (95.1%) | 75 (96.2%) | 91 (91.9%) | 67 (94.4%) | 68 (94.4%) |
| **NSCLC histology NOS** | <5 | <5 | <5 | <5 | <5 | <5 | <5 | <5 |
| **Ethnicity** |  |  |  |  |  |  |  |  |
| **White** | 34 (63%) | 50 (68.5%) | 54 (77.1%) | 60 (74.1%) | 50 (64.1%) | 75 (75.8%) | 47 (66.2%) | 49 (68.1%) |
| **Black or African American** | 7 (13%) | 9 (12.3%) | <5 | 6 (7.4%) | 8 (10.3%) | 6 (6.1%) | 5 (7%) | <5 |
| **Others** | 13 (24.1%) | 14 (19.2%) | 13 (18.6%) | 15 (18.5%) | 20 (25.6%) | 18 (18.2%) | 19 (26.8%) | 21 (29.2%) |
| **Treatment, n(%)**** |  |  |  |  |  |  |  |  |
| **Any ALKi use** |  |  |  |  |  |  |  |  |
| **1^st^ gen ALKi and 2^nd^/3^rd^ gen ALKi** | 5 (9.3%) | 21 (28.8%) | 28 (40%) | 40 (49.4%) | 30 (38.5%) | 18 (18.2%) | <5 | <5 |
| **1^st^ gen ALKi only** | 45 (83.3%) | 47 (64.4%) | 36 (51.4%) | 28 (34.6%) | 32 (41%) | 20 (20.2%) | <5 | <5 |
| **2^nd^/3^rd^ gen ALKi only** | <5 | <5 | <5 | <5 | 7 (9%) | 47 (47.5%) | 55 (77.5%) | 56 (77.8%) |
| **No ALKi use** | <5 | 5 (6.8%) | <5 | 9 (11.1%) | 9 (11.5%) | 14 (14.1%) | 11 (15.5%) | 14 (19.4%) |
|  | **EGFR+ mNSCLC patients** | | | | | | | |
| **Age, Mean (SD)** | 65.8 (10.48) | 65.94 (10.37) | 67.57 (10.14) | 67.1 (10.41) | 68.33 (10.35) | 68.04 (10.66) | 68.46 (10.17) | 69.17 (10.11) |
| **Sex, n(%)** |  |  |  |  |  |  |  |  |
| **Male** | 64 (40.3%) | 68 (32.5%) | 101 (35.7%) | 97 (32.6%) | 117 (34.8%) | 122 (35.1%) | 135 (32.8%) | 130 (31%) |
| **Female** | 95 (59.7%) | 141 (67.5%) | 182 (64.3%) | 201 (67.4%) | 219 (65.2%) | 226 (64.9%) | 277 (67.2%) | 289 (69%) |
| **Smoking status, n(%)** |  |  |  |  |  |  |  |  |
| **Smoker** | 79 (50.3%) | 112 (53.8%) | 146 (52.1%) | 143 (48%) | 170 (50.7%) | 175 (50.4%) | 219 (53.2%) | 210 (50.1%) |
| **Non-smoker** | 78 (49.7%) | 96 (46.2%) | 134 (47.9%) | 155 (52%) | 165 (49.3%) | 172 (49.6%) | 193 (46.8%) | 209 (49.9%) |
| **Stage, n(%)** |  |  |  |  |  |  |  |  |
| **0/I** | 10 (6.3%) | 10 (4.8%) | 21 (7.4%) | 22 (7.4%) | 26 (7.7%) | 23 (6.6%) | 28 (6.8%) | 38 (9.1%) |
| **II/III** | 22 (13.8%) | 37 (17.7%) | 45 (15.9%) | 42 (14.1%) | 54 (16.1%) | 51 (14.7%) | 62 (15%) | 68 (16.2%) |
| **IV** | 127 (79.9%) | 162 (77.5%) | 217 (76.7%) | 234 (78.5%) | 256 (76.2%) | 274 (78.7%) | 322 (78.2%) | 313 (74.7%) |
| **Time to 1L treatment, Median (IQR)** | 1.18 (0.69 - 1.89) | 1.08 (0.66 - 1.84) | 1.08 (0.66 - 1.69) | 1.12 (0.72 - 1.63) | 1.12 (0.69 - 1.84) | 1.12 (0.76 - 1.68) | 1.12 (0.72 - 1.61) | 1.08 (0.72 - 1.54) |
| **Histology** |  |  |  |  |  |  |  |  |
| **Squamous cell carcinoma** | 5 (3.1%) | <5 | <5 | <5 | 7 (2.1%) | 10 (2.9%) | 13 (3.2%) | 10 (2.4%) |
| **Non-squamous cell carcinoma** | 149 (93.7%) | 204 (97.6%) | 280 (98.9%) | 291 (97.7%) | 325 (96.7%) | 333 (95.7%) | 392 (95.1%) | 398 (95%) |
| **NSCLC histology NOS** | 5 (3.1%) | <5 | <5 | <5 | <5 | 5 (1.4%) | 7 (1.7%) | 11 (2.6%) |
| **Ethnicity** |  |  |  |  |  |  |  |  |
| **White** | 97 (61%) | 140 (67%) | 195 (68.9%) | 209 (70.1%) | 226 (67.3%) | 228 (65.5%) | 262 (63.6%) | 258 (61.6%) |
| **Black or African American** | 16 (10.1%) | 18 (8.6%) | 24 (8.5%) | 26 (8.7%) | 23 (6.8%) | 30 (8.6%) | 36 (8.7%) | 36 (8.6%) |
| **Others** | 46 (28.9%) | 51 (24.4%) | 64 (22.6%) | 63 (21.1%) | 87 (25.9%) | 90 (25.9%) | 114 (27.7%) | 125 (29.8%) |
| **Treatment, n(%)**** |  |  |  |  |  |  |  |  |
| **Any EGFR use** |  |  |  |  |  |  |  |  |
| **1^st^/2^nd^ gen EGFRi and 3^rd^ gen EGFRi** | <5 | 10 (4.8%) | 26 (9.2%) | 45 (15.1%) | 78 (23.2%) | 83 (23.9%) | 53 (12.9%) | 14 (3.3%) |
| **1^st^/2^nd^ gen EGFRi only** | 135 (84.9%) | 164 (78.5%) | 226 (79.9%) | 210 (70.5%) | 213 (63.4%) | 170 (48.9%) | 59 (14.3%) | 30 (7.2%) |
| **3^rd^ gen EGFRi only** | <5 | <5 | <5 | <5 | 13 (3.9%) | 50 (14.4%) | 235 (57%) | 291 (69.5%) |
| **No EGFRi use** | 21 (13.2%) | 34 (16.3%) | 31 (11%) | 41 (13.8%) | 32 (9.5%) | 45 (12.9%) | 65 (15.8%) | 84 (20%) |

Notes: *use of FDA approved immunotherapies for 1L or 2L metastatic NSCLC and off-label/trial-based use of non-FDA approved immunotherapies in 1L or 2L metastatic NSCLC categorised separately given expected differential impact on survival over time **treatment data for these aggregated by treatment type and not stratified by line of therapy given small numbers. SD, standard deviation; IQR, interquartile range; 1L, first line therapy; 2L second line therapy

Supplementary Table 3. Hazard ratios for overall survival among mNSCLC patients, stratified by biomarker status.

|  | **Primary analysis** | **Post-hoc analysis** |
| --- | --- | --- |
|  | **HR (95% CI)** | **HR (95% CI)** |
|  | **Non biomarker positive mNSCLC patients** | |
| **Calendar year (ref. 2012)** |  |  |
| **2012 (ref)** |  |  |
| **2013** | 0.98 (0.93 - 1.04) | 0.97 (0.91 - 1.03) |
| **2014** | 1.02 (0.96 - 1.08) | 0.94 (0.89 – 1.00) |
| **2015** | 1.07 (1.01 - 1.14) | 0.95 (0.89 - 1.01) |
| **2016** | 1.16 (1.09 - 1.23) | 0.96 (0.90 - 1.02) |
| **2017** | 1.20 (1.13 - 1.28) | 0.98 (0.92 - 1.05) |
| **2018** | 1.24 (1.16 - 1.33) | 1.02 (0.94 - 1.09) |
| **2019** | 1.27 (1.18 - 1.37) | 0.99 (0.91 - 1.07) |
| **Age** | 1.01 (1.01 - 1.01) | 1.01 (1.00 - 1.01) |
| **Sex** |  |  |
| **Male** | 1.20 (1.17 - 1.24) | 1.21 (1.17 - 1.25) |
| **Female (ref)** |  |  |
| **Smoking status (ref. non-smoker)** |  |  |
| **Smoker** | 1.40 (1.33 - 1.48) | 1.33 (1.26 - 1.40) |
| **Non-smoker (ref)** |  |  |
| **Stage** |  |  |
| **0/I (ref)** |  |  |
| **II/III** | 1.11 (1.04 - 1.18) | 1.13 (1.06 - 1.20) |
| **IV** | 1.69 (1.60 - 1.78) | 1.75 (1.65 - 1.86) |
| **Time to 1L treatment** | 0.95 (0.94 - 0.95) | 0.93 (0.92 - 0.93) |
| **Histology** |  |  |
| **Squamous cell carcinoma** | 1.14 (1.10 - 1.18) | 1.11 (1.08 - 1.16) |
| **Non-squamous cell carcinoma (ref)** |  |  |
| **NSCLC histology NOS** | 1.46 (1.37 - 1.56) | 1.41 (1.32 - 1.52) |
| **Ethnicity** |  |  |
| **White (ref)** |  |  |
| **Black or African American** | 0.89 (0.85 - 0.94) | 0.90 (0.86 - 0.95) |
| **Others** | 0.86 (0.82 - 0.90) | 0.88 (0.84 - 0.92) |
| **Treatment*** |  |  |
| **Approved and non-approved immunotherapy** | 0.32 (0.26 - 0.40) |  |
| **1L approved IT; 2L non-approved IT** |  | 0.22 (0.12 - 0.41) |
| **1L non-approved IT; 2L approved IT** |  | 0.25 (0.19 - 0.31) |
| **Approved immunotherapies only** | 0.57 (0.55 - 0.59) |  |
| **1L approved IT; 2L approved IT** |  | 0.22 (0.19 - 0.25) |
| **1L approved IT; 2L Non-IT therapy** |  | 0.31 (0.29 - 0.34) |
| **1L approved IT; 2L no therapy** |  | 0.60 (0.56 - 0.64) |
| **1L Non-IT therapy; 2L approved IT** |  | 0.37 (0.35 - 0.38) |
| **Non-approved immunotherapies only** | 0.59 (0.55 - 0.63) |  |
| **1L non-approved IT; 2L non-approved IT** |  | 0.46 (0.20 - 1.05) |
| **1L non-approved IT; 2L Non-IT therapy** |  | 0.57 (0.51 - 0.64) |
| **1L non-approved IT; 2L no therapy** |  | 0.87 (0.79 - 0.96) |
| **1L Non-IT therapy; 2L non-approved IT** |  | 0.16 (0.14 - 0.19) |
| **No immunotherapy** | Reference |  |
| **1L Non-IT therapy; 2L Non-IT therapy** |  | 0.40 (0.39 - 0.42) |
| **1L Non-IT therapy; 2L no therapy** |  | Reference |
|  | **ALK+ mNSCLC patients** | |
| **Calendar year (ref. 2012)** |  |  |
| **2012 (ref)** |  |  |
| **2013** | 0.87 (0.55 - 1.40) | 1.04 (0.64 - 1.66) |
| **2014** | 0.93 (0.57 - 1.51) | 1.31 (0.80 - 2.14) |
| **2015** | 0.92 (0.59 - 1.43) | 1.45 (0.90 - 2.35) |
| **2016** | 1.00 (0.63 - 1.59) | 1.42 (0.87 - 2.34) |
| **2017** | 0.62 (0.39 - 0.99) | 1.20 (0.70 - 2.05) |
| **2018** | 0.45 (0.25 - 0.80) | 1.13 (0.54 - 2.37) |
| **2019** | 0.41 (0.21 - 0.79) | 1.04 (0.48 - 2.24) |
| **Age** | 1.01 (1.00 - 1.02) | 1.01 (1.00 - 1.02) |
| **Sex** |  |  |
| **Male** | 1.04 (0.82 - 1.32) | 1.08 (0.85 - 1.37) |
| **Female (ref)** |  |  |
| **Smoking status (ref. non-smoker)** |  |  |
| **Smoker** | 1.26 (0.98 - 1.63) | 1.26 (0.98 - 1.64) |
| **Non-smoker (ref)** |  |  |
| **Stage** |  |  |
| **0/I (ref)** |  |  |
| **II/III** | 1.37 (0.77 - 2.42) | 1.26 (0.68 - 2.33) |
| **IV** | 2.82 (1.71 - 4.66) | 2.75 (1.59 - 4.75) |
| **Time to 1L treatment** | 0.96 (0.94 - 0.99) | 0.98 (0.95 – 1.00) |
| **Histology** |  |  |
| **Squamous cell carcinoma** | 1.63 (0.81 - 3.27) | 1.54 (0.78 - 3.04) |
| **Non-squamous cell carcinoma (ref)** |  |  |
| **NSCLC histology NOS** | 1.39 (0.68 - 2.83) | 1.39 (0.69 - 2.78) |
| **Ethnicity** |  |  |
| **White (ref)** |  |  |
| **Black or African American** | 0.94 (0.60 - 1.49) | 0.90 (0.56 - 1.43) |
| **Others** | 0.71 (0.53 - 0.96) | 0.74 (0.55 - 0.99) |
| **Treatment**** |  |  |
| **Any ALKi use** | 0.50 (0.33 - 0.76) |  |
| **1^st^ gen ALKi and 2^nd^/3^rd^ gen ALKi** |  | 0.41 (0.25 - 0.67) |
| **1^st^ gen ALKi only** |  | 0.94 (0.57 - 1.55) |
| **2^nd^/3^rd^ gen ALKi only** |  | 0.29 (0.17 - 0.47) |
| **No ALKi use** | Reference | Reference |
|  | **EGFR+ mNSCLC patients** | |
| **Calendar year (ref. 2012)** |  |  |
| **2012 (ref)** |  |  |
| **2013** | 0.90 (0.72 - 1.13) | 0.94 (0.75 - 1.18) |
| **2014** | 0.95 (0.77 - 1.17) | 1.02 (0.82 - 1.26) |
| **2015** | 1.01 (0.82 - 1.25) | 1.14 (0.92 - 1.42) |
| **2016** | 1.00 (0.81 - 1.23) | 1.20 (0.97 - 1.49) |
| **2017** | 0.98 (0.79 - 1.21) | 1.25 (1.00 - 1.56) |
| **2018** | 0.87 (0.70 - 1.09) | 1.20 (0.92 - 1.55) |
| **2019** | 0.99 (0.77 - 1.28) | 1.34 (1.00 - 1.81) |
| **Age** | 1.02 (1.01 - 1.03) | 1.02 (1.01 - 1.02) |
| **Sex** |  |  |
| **Male** | 1.11 (0.99 - 1.24) | 1.11 (0.99 - 1.24) |
| **Female (ref)** |  |  |
| **Smoking status (ref. non-smoker)** |  |  |
| **Smoker** | 1.31 (1.17 - 1.45) | 1.31 (1.18 - 1.46) |
| **Non-smoker (ref)** |  |  |
| **Stage** |  |  |
| **0/I (ref)** |  |  |
| **II/III** | 1.11 (0.85 - 1.45) | 1.10 (0.85 - 1.44) |
| **IV** | 1.72 (1.35 - 2.18) | 1.75 (1.38 - 2.21) |
| **Time to 1L treatment** | 0.95 (0.93 - 0.97) | 0.95 (0.93 - 0.97) |
| **Histology** |  |  |
| **Squamous cell carcinoma** | 1.61 (1.17 - 2.23) | 1.52 (1.10 - 2.11) |
| **Non-squamous cell carcinoma (ref)** |  |  |
| **NSCLC histology NOS** | 1.22 (0.80 - 1.87) | 1.27 (0.82 - 1.97) |
| **Ethnicity** |  |  |
| **White (ref)** |  |  |
| **Black or African American** | 0.97 (0.80 - 1.18) | 0.93 (0.76 - 1.14) |
| **Others** | 0.84 (0.74 - 0.96) | 0.83 (0.72 - 0.94) |
| **Treatment**** |  |  |
| **Any EGFR use** | 0.55 (0.47 - 0.65) |  |
| **1^st^/2^nd^ gen EGFRi and 3^rd^ gen EGFRi** |  | 0.31 (0.25 - 0.38) |
| **1^st^/2^nd^ gen EGFRi only** |  | 0.69 (0.58 - 0.82) |
| **3^rd^ gen EGFRi only** |  | 0.48 (0.38 - 0.60) |
| **No EGFRi use** | Reference | Reference |

Notes: *use of FDA approved immunotherapies for 1L or 2L metastatic NSCLC and off-label/trial-based use of non-FDA approved immunotherapies in 1L or 2L metastatic NSCLC categorised separately given expected differential impact on survival over time **treatment data for these aggregated by treatment type and not stratified by line of therapy given small numbers. SD, standard deviation; IQR, interquartile range; 1L, first line therapy; 2L second line therapy
