# Supplementary material for: The value of innovation: association between improvements in survival of advanced and metastatic non-small cell lung cancer and targeted and immunotherapy
Source: BMC Med. 2021 Sep 15;19:209. doi: 10.1186/s12916-021-02070-w (PMC8442434; doi:10.1186/s12916-021-02070-w)
Supplement: Supplementary file 3 — Additional file 3. Sensitivity analysis plots. Description: Plots of the hazard ratios from both sensitivity analyses for each population and for both the main analysis and post hoc analysis. It also includes a table of the additional covariates used in the sensitivity analysis stratified by year. [file 12916_2021_2070_MOESM3_ESM.docx]

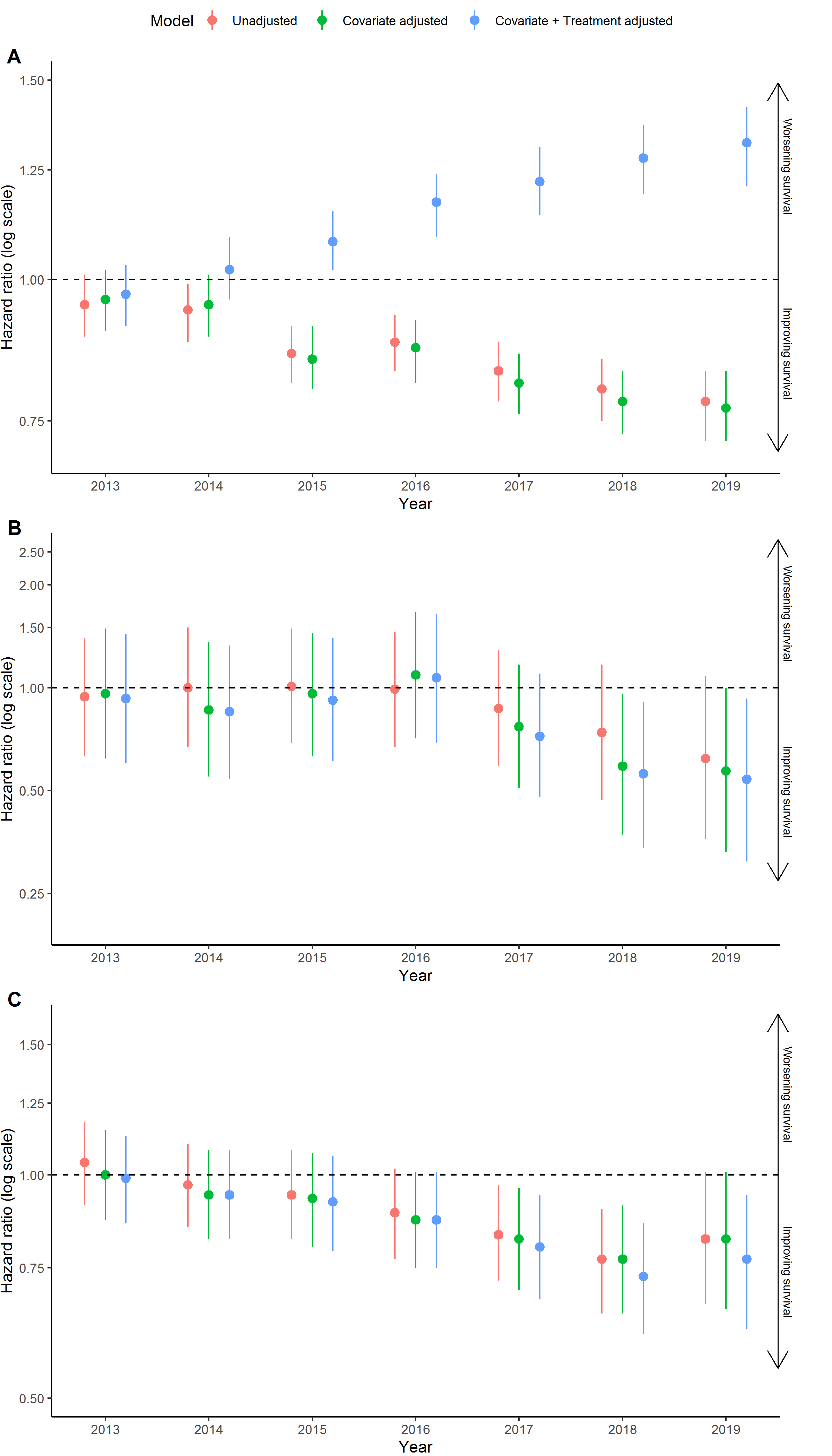


Supplementary Figure 2. Hazard ratios for death in non-oncogene positive (A) advanced and/or metastatic NSCLC (B) ALK-positive and (C) EGFR-positive patients in years 2013 to 2019 relative to 2012, unadjusted, adjusting only for differences in baseline characteristics and adjusting for both baseline characteristics and (A) immunotherapy use (B) ALKi use and (C) EGFRi use for the sensitivity analysis for misclassification of the population.


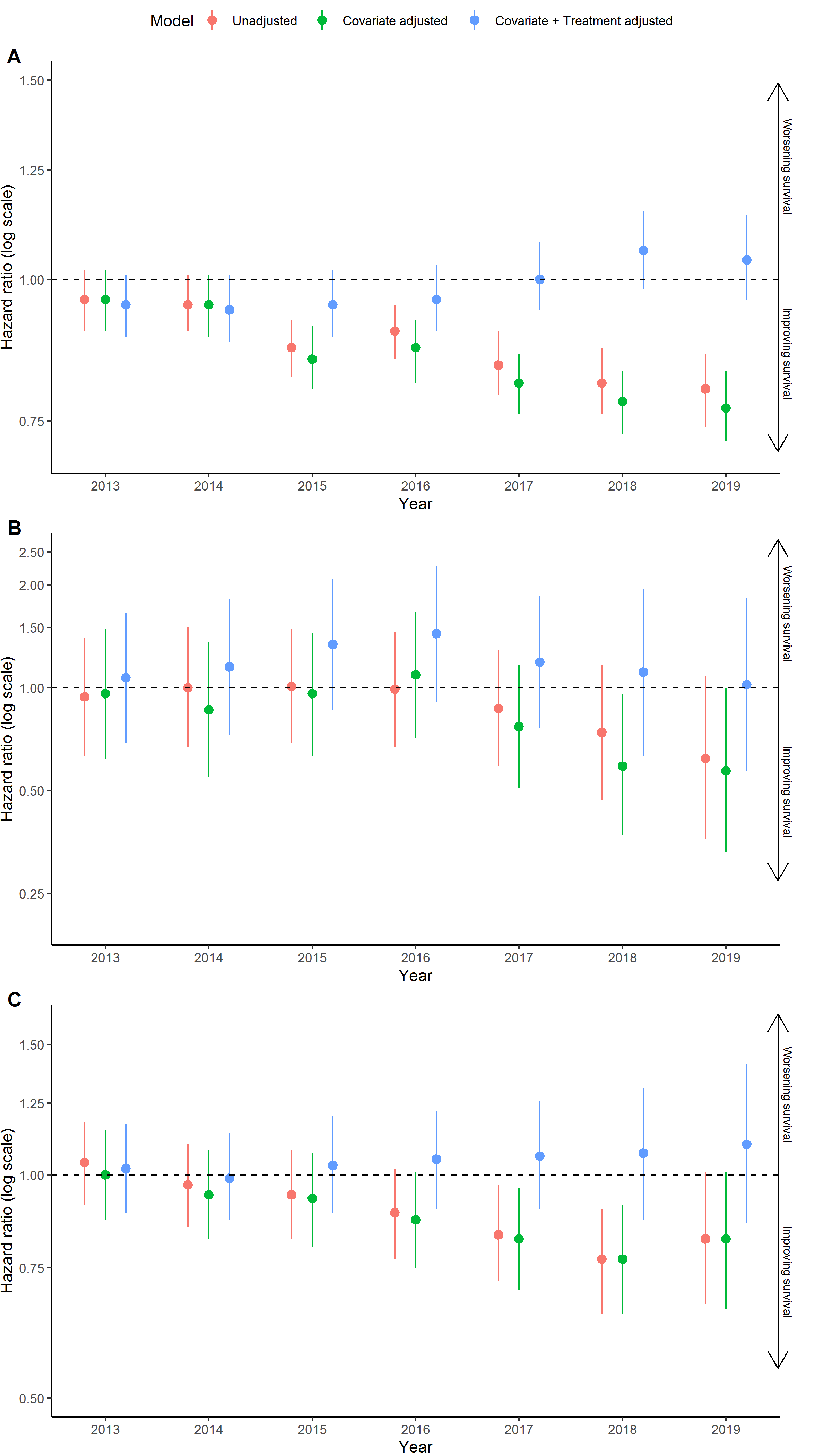


Supplementary Figure 3. Hazard ratios for death in non-oncogene positive (A) advanced and/or metastatic NSCLC (B) ALK-positive and (C) EGFR-positive patients in years 2013 to 2019 relative to 2012, unadjusted, adjusting only for differences in baseline characteristics and adjusting for both baseline characteristics and (A) first and/or second line immunotherapy, (B) first and/or second/third generation ALKi use, (C) first/second and/or third generation EGFRi use for the sensitivity analysis for misclassification of the population.


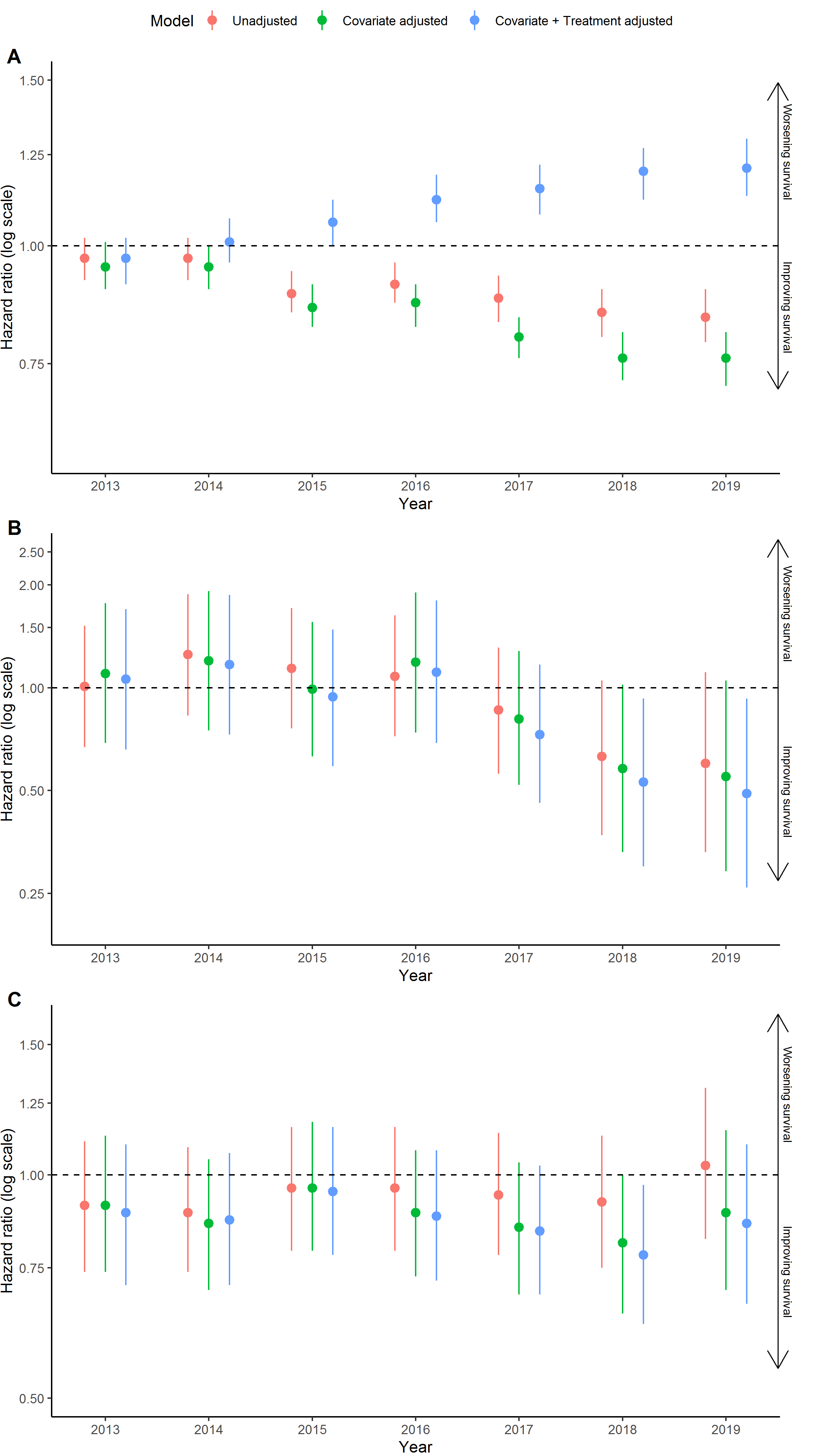


Supplementary Figure 4. Hazard ratios for death in non-oncogene positive (A) advanced and/or metastatic NSCLC (B) ALK-positive and (C) EGFR-positive patients in years 2013 to 2019 relative to 2012, unadjusted, adjusting only for differences in baseline characteristics and adjusting for both baseline characteristics and (A) immunotherapy use (B) ALKi use and (C) EGFRi use for the sensitivity analysis including a missingness category for variables with missing data and additionally adjusting for practice type, insurance type, ECOG, and metastatic site.


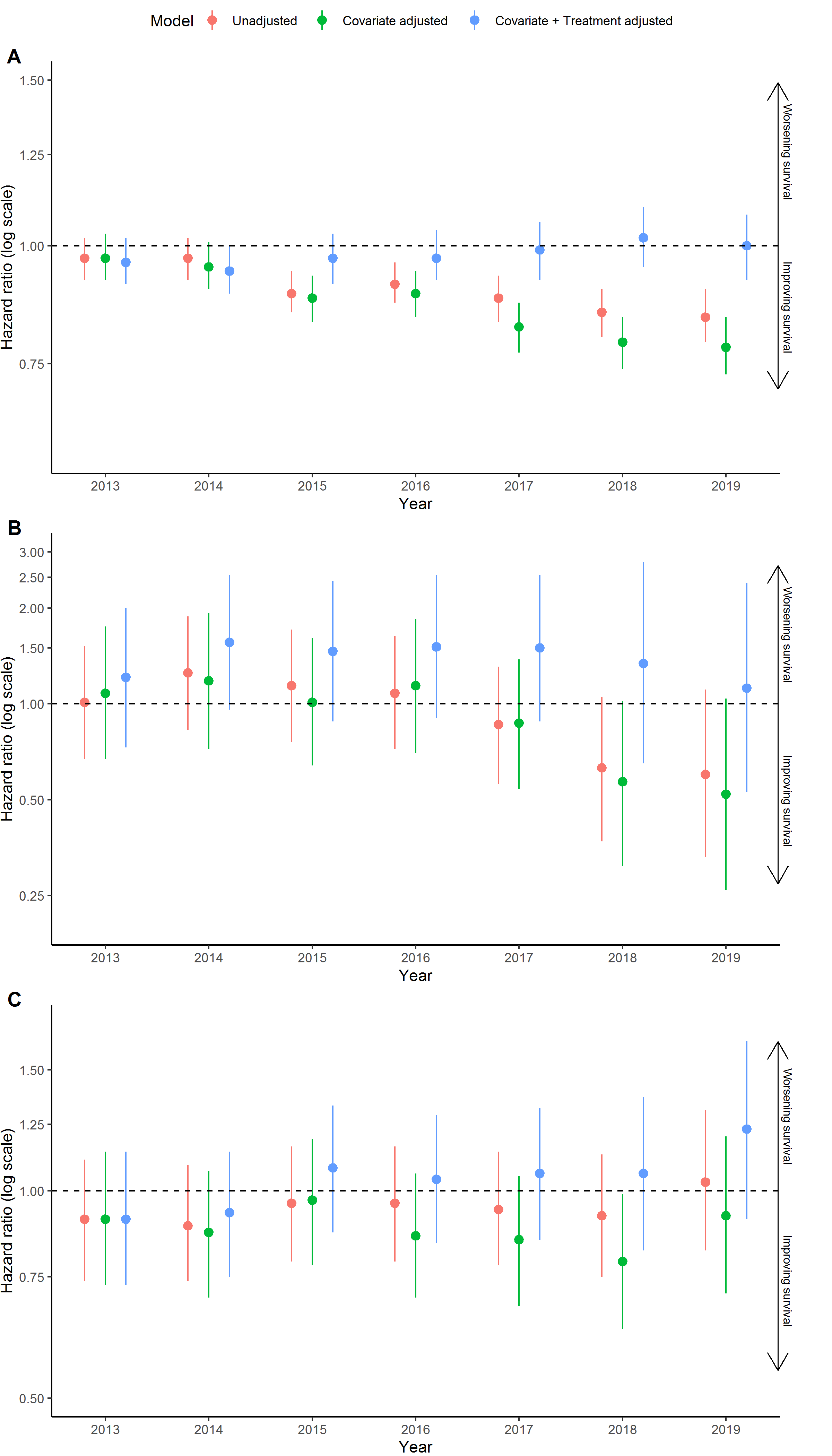


Supplementary Figure 5. Hazard ratios for death in non-oncogene positive (A) advanced and/or metastatic NSCLC (B) ALK-positive and (C) EGFR-positive patients in years 2013 to 2019 relative to 2012, unadjusted, adjusting only for differences in baseline characteristics and adjusting for both baseline characteristics and (A) first and/or second line immunotherapy, (B) first and/or second/third generation ALKi use, (C) first/second and/or third generation EGFRi use for the sensitivity analysis including a missingness category for variables with missing data and additionally adjusting for practice type, insurance type, ECOG, and metastatic site.

*Supplementary Table 4. Additional covariates that were adjusted for in the sensitivity analysis considering missingness and peripheral covariates stratified by year.*

|  | **2012** | **2013** | **2014** | **2015** | **2016** | **2017** | **2018** | **2019** |
| --- | --- | --- | --- | --- | --- | --- | --- | --- |
|  | **Non biomarker positive mNSCLC patients** | | | | | | | |
| **Practice type** |  |  |  |  |  |  |  |  |
| Academic | 146 (4.8%) | 267 (7.6%) | 314 (7.7%) | 366 (8.5%) | 350 (7.7%) | 367 (8%) | 380 (8.6%) | 325 (8.3%) |
| Community | 2883 (95.2%) | 3243 (92.4%) | 3776 (92.3%) | 3959 (91.5%) | 4177 (92.3%) | 4228 (92%) | 4059 (91.4%) | 3609 (91.7%) |
| **Insurance type** |  |  |  |  |  |  |  |  |
| Medicaid/Medicare | 635 (21%) | 739 (21.1%) | 958 (23.4%) | 1038 (24%) | 1080 (23.9%) | 1074 (23.4%) | 1021 (23%) | 861 (21.9%) |
| Other Government Program/Other Payer | 266 (8.8%) | 356 (10.1%) | 403 (9.9%) | 464 (10.7%) | 520 (11.5%) | 537 (11.7%) | 474 (10.7%) | 492 (12.5%) |
| Patient Assistance Program/Self Pay | 40 (1.3%) | 70 (2%) | 69 (1.7%) | 83 (1.9%) | 81 (1.8%) | 93 (2%) | 99 (2.2%) | 80 (2%) |
| Commercial Health Plan | 1294 (42.7%) | 1527 (43.5%) | 1832 (44.8%) | 2078 (48%) | 2244 (49.6%) | 2367 (51.5%) | 2408 (54.2%) | 2171 (55.2%) |
| Missing | 794 (26.2%) | 818 (23.3%) | 828 (20.2%) | 662 (15.3%) | 602 (13.3%) | 524 (11.4%) | 437 (9.8%) | 330 (8.4%) |
| **ECOG** |  |  |  |  |  |  |  |  |
| 0-1 | 772 (25.5%) | 1088 (31%) | 1404 (34.3%) | 1580 (36.5%) | 1927 (42.6%) | 2108 (45.9%) | 2174 (49%) | 1995 (50.7%) |
| 2-4 | 240 (7.9%) | 347 (9.9%) | 492 (12%) | 499 (11.5%) | 591 (13.1%) | 690 (15%) | 684 (15.4%) | 656 (16.7%) |
| Missing/not recorded | 2017 (66.6%) | 2075 (59.1%) | 2194 (53.6%) | 2246 (51.9%) | 2009 (44.4%) | 1797 (39.1%) | 1581 (35.6%) | 1283 (32.6%) |
| **Metastases type** |  |  |  |  |  |  |  |  |
| Liver | 9 (0.3%) | 9 (0.3%) | 22 (0.5%) | 25 (0.6%) | 22 (0.5%) | 21 (0.5%) | 33 (0.7%) | 18 (0.5%) |
| CNS | 266 (8.8%) | 352 (10%) | 402 (9.8%) | 421 (9.7%) | 512 (11.3%) | 564 (12.3%) | 562 (12.7%) | 510 (13%) |
| Liver + CNS | 6 (0.2%) | 5 (0.1%) | 6 (0.1%) | 14 (0.3%) | 7 (0.2%) | 15 (0.3%) | 6 (0.1%) | 5 (0.1%) |
| Other (without liver/CNS) | 1238 (40.9%) | 1526 (43.5%) | 1797 (43.9%) | 1896 (43.8%) | 1988 (43.9%) | 2017 (43.9%) | 1935 (43.6%) | 1786 (45.4%) |
| Missing/not recorded | 1510 (49.9%) | 1618 (46.1%) | 1863 (45.6%) | 1969 (45.5%) | 1998 (44.1%) | 1978 (43%) | 1903 (42.9%) | 1615 (41.1%) |
|  | **ALK+ mNSCLC patients** | | | | | | | |
| **Practice type** |  |  |  |  |  |  |  |  |
| Academic | 8 (13.6%) | 12 (14.3%) | 16 (19%) | 14 (15.9%) | 7 (7.5%) | 16 (14.4%) | 13 (15.7%) | 13 (15.5%) |
| Community | 51 (86.4%) | 72 (85.7%) | 68 (81%) | 74 (84.1%) | 86 (92.5%) | 95 (85.6%) | 70 (84.3%) | 71 (84.5%) |
| **Insurance type** |  |  |  |  |  |  |  |  |
| Medicaid/Medicare | 11 (18.6%) | 9 (10.7%) | 19 (22.6%) | 17 (19.3%) | 16 (17.2%) | 16 (14.4%) | 8 (9.6%) | 9 (10.7%) |
| Other Government Program/Other Payer | 6 (10.2%) | 10 (11.9%) | 11 (13.1%) | 7 (8%) | 13 (14%) | 14 (12.6%) | 12 (14.5%) | 18 (21.4%) |
| Patient Assistance Program/Self Pay | <5 | <5 | <5 | <5 | <5 | <5 | <5 | <5 |
| Commercial Health Plan | 20 (33.9%) | 35 (41.7%) | 42 (50%) | 52 (59.1%) | 49 (52.7%) | 66 (59.5%) | 52 (62.7%) | 53 (63.1%) |
| Missing | 22 (37.3%) | 28 (33.3%) | 11 (13.1%) | 9 (10.2%) | 14 (15.1%) | 13 (11.7%) | 11 (13.3%) | <5 |
| **ECOG** |  |  |  |  |  |  |  |  |
| 0-1 | 11 (18.6%) | 23 (27.4%) | 25 (29.8%) | 29 (33%) | 43 (46.2%) | 54 (48.6%) | 38 (45.8%) | 49 (58.3%) |
| 2-4 | <5 | 5 (6%) | 9 (10.7%) | 10 (11.4%) | 11 (11.8%) | 8 (7.2%) | 9 (10.8%) | 10 (11.9%) |
| Missing/not recorded | 44 (74.6%) | 56 (66.7%) | 50 (59.5%) | 49 (55.7%) | 39 (41.9%) | 49 (44.1%) | 36 (43.4%) | 25 (29.8%) |
| **Metastases type** |  |  |  |  |  |  |  |  |
| Liver | <5 | <5 | <5 | <5 | <5 | <5 | <5 | <5 |
| CNS | 12 (20.3%) | 7 (8.3%) | 10 (11.9%) | 13 (14.8%) | 15 (16.1%) | 15 (13.5%) | 9 (10.8%) | 15 (17.9%) |
| Liver + CNS | <5 | <5 | <5 | <5 | <5 | <5 | <5 | <5 |
| Other (without liver/CNS) | 26 (44.1%) | 34 (40.5%) | 35 (41.7%) | 40 (45.5%) | 35 (37.6%) | 51 (45.9%) | 42 (50.6%) | 38 (45.2%) |
| Missing/not recorded | 21 (35.6%) | 43 (51.2%) | 38 (45.2%) | 34 (38.6%) | 43 (46.2%) | 45 (40.5%) | 31 (37.3%) | 30 (35.7%) |
|  | **EGFR+ mNSCLC patients** | | | | | | | |
| **Practice type** |  |  |  |  |  |  |  |  |
| Academic | 17 (9.6%) | 22 (9%) | 32 (9.9%) | 28 (8.1%) | 38 (9.5%) | 32 (7.4%) | 42 (8.7%) | 49 (9.6%) |
| Community | 161 (90.4%) | 222 (91%) | 292 (90.1%) | 318 (91.9%) | 361 (90.5%) | 398 (92.6%) | 443 (91.3%) | 461 (90.4%) |
| **Insurance type** |  |  |  |  |  |  |  |  |
| Medicaid/Medicare | 35 (19.7%) | 64 (26.2%) | 73 (22.5%) | 66 (19.1%) | 67 (16.8%) | 91 (21.2%) | 98 (20.2%) | 89 (17.5%) |
| Other Government Program/Other Payer | 22 (12.4%) | 23 (9.4%) | 36 (11.1%) | 33 (9.5%) | 50 (12.5%) | 57 (13.3%) | 69 (14.2%) | 71 (13.9%) |
| Patient Assistance Program/Self Pay | <5 | 5 (2%) | 5 (1.5%) | 8 (2.3%) | 8 (2%) | 6 (1.4%) | 9 (1.9%) | 10 (2%) |
| Commercial Health Plan | 70 (39.3%) | 97 (39.8%) | 151 (46.6%) | 164 (47.4%) | 205 (51.4%) | 224 (52.1%) | 259 (53.4%) | 302 (59.2%) |
| Missing | 49 (27.5%) | 55 (22.5%) | 59 (18.2%) | 75 (21.7%) | 69 (17.3%) | 52 (12.1%) | 50 (10.3%) | 38 (7.5%) |
| **ECOG** |  |  |  |  |  |  |  |  |
| 0-1 | 32 (18%) | 62 (25.4%) | 94 (29%) | 128 (37%) | 171 (42.9%) | 227 (52.8%) | 245 (50.5%) | 269 (52.7%) |
| 2-4 | <5 | 16 (6.6%) | 33 (10.2%) | 25 (7.2%) | 46 (11.5%) | 51 (11.9%) | 70 (14.4%) | 65 (12.7%) |
| Missing/not recorded | 142 (79.8%) | 166 (68%) | 197 (60.8%) | 193 (55.8%) | 182 (45.6%) | 152 (35.3%) | 170 (35.1%) | 176 (34.5%) |
| **Metastases type** |  |  |  |  |  |  |  |  |
| Liver | <5 | <5 | <5 | <5 | <5 | <5 | <5 | <5 |
| CNS | 16 (9%) | 32 (13.1%) | 51 (15.7%) | 46 (13.3%) | 64 (16%) | 73 (17%) | 94 (19.4%) | 97 (19%) |
| Liver + CNS | <5 | <5 | <5 | <5 | <5 | <5 | <5 | <5 |
| Other (without liver/CNS) | 67 (37.6%) | 103 (42.2%) | 132 (40.7%) | 154 (44.5%) | 167 (41.9%) | 174 (40.5%) | 201 (41.4%) | 223 (43.7%) |
| Missing/not recorded | 94 (52.8%) | 109 (44.7%) | 138 (42.6%) | 142 (41%) | 165 (41.4%) | 182 (42.3%) | 189 (39%) | 186 (36.5%) |
